# Mimickers of Systemic Lupus Erythematosus: Case Series and Literature Overview

**DOI:** 10.3390/jcm14197070

**Published:** 2025-10-07

**Authors:** Kaj L. Roest, A. (Liesbeth) E. Hak, Ester M. M. van Leeuwen, Godelieve J. de Bree, Arjan J. Kwakernaak

**Affiliations:** 1 Department of Internal Medicine, Division of Clinical Immunology and Allergy, Center for Immunodeficiency and Immune Dysregulation Amsterdam, Amsterdam Institute for Immunology and Infectious Diseases, Amsterdam University Medical Center, University of Amsterdam, 1105 AZ Amsterdam, The Netherlands; k.l.roest@amsterdamumc.nl (K.L.R.);; 2 Department of Laboratory Medicine, Laboratory Specialized Diagnostics and Research, Section Medical Immunology, Department of Experimental Immunology, Amsterdam Institute for Immunology and Infectious Diseases, Amsterdam University Medical Center, University of Amsterdam, 1105 AZ Amsterdam, The Netherlands; 3 Department of Internal Medicine, Division of Infectious Diseases, Amsterdam University Medical Center, University of Amsterdam, 1105 AZ Amsterdam, The Netherlands; 4 Department of Internal Medicine, Division of Nephrology, Amsterdam University Medical Center, University of Amsterdam, 1105 AZ Amsterdam, The Netherlands

**Keywords:** systemic lupus erythematosus, SLE-mimicking disease, inborn errors of immunity, primary immunodeficiency, monogenic interferonopathy, Aicardi–Goutières syndrome, neuromyelitis optica spectrum disease

## Abstract

Systemic lupus erythematosus (SLE) is an autoimmune disease characterized by a broad and varied clinical presentation. In the absence of definite diagnostic criteria, establishing an SLE diagnosis remains challenging. This case series illustrates that other diseases, such as primary immunodeficiencies and monogenic interferonopathies, can closely mimic SLE, even in the presence of its typical serological markers. Recognizing these disease mimickers is crucial to avoid premature conclusions in clinical evaluation and to ensure the initiation of appropriate therapy. Especially in cases of atypical presentation, unusual disease progression, or resistance to standard therapy, alternative diagnoses should be considered. In this overview, we discuss the diagnostic approach for patients with SLE-like manifestations and provide a comprehensive review of diseases that may mimic SLE.

## 1. Introduction

Systemic lupus erythematosus (SLE) is a systemic autoimmune disease renowned for its diverse clinical spectrum, often referred to as the ‘great imitator’. This moniker highlights the significant diagnostic challenges it presents. First, diagnosis relies not on a single test but on a combination of specific clinical and laboratory findings alongside a positive antinuclear antibody (ANA) test. The gradual and non-specific onset of symptoms frequently leads to a diagnostic delay that can result in irreversible organ damage. Compounding this complexity, a positive ANA test, while a hallmark of SLE, is also observed in healthy individuals and patients with other autoimmune diseases, such as systemic sclerosis and Sjögren’s disease. Furthermore, several specific conditions, including primary immunodeficiencies and monogenic interferonopathies, as well as infections and (hematological) malignancies, can mimic SLE, complicating the diagnostic process and further delaying correct identification. This paper describes three patients initially diagnosed with SLE whose final diagnosis revealed a distinct etiology for their symptoms, resulting in important modifications to their treatment regimen. Moreover, we provide a diagnostic framework to aid in the robust identification of these SLE-mimicking diseases.

## 2. Case Series

**Figure 1:**
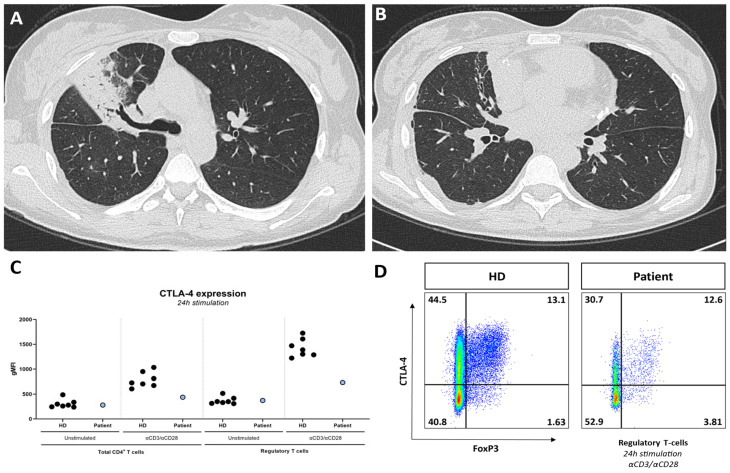
CT-scan showing lobar pneumonia (**A**) and focal bronchiectasis (**B**); (**C**) CTLA-4 expression on CD4^+^ T-cells and regulatory T-cells, unstimulated and after 24 h stimulation with aCD3/aCD28, in the patient and healthy donors (HDs), showing decreased CTLA-4 expression on CD4^+^ T-cells and regulatory T-cells during stimulated conditions in the patient compared with HDs; (**D**) decreased CTLA-4 expression FoxP3^+^ regulatory T-cells demonstrated through flow cytometry.

Case 1: Our first case is a 45-year-old woman diagnosed at 19 years of age with systemic lupus erythematosus (SLE). The diagnosis was based on a new-onset Raynaud’s phenomenon, oral ulceration, non-scaring alopecia, serositis (pericarditis and pleuritis), symmetric polyarthritis, autoimmune hemolytic anemia (AIHA; direct antiglobulin testing (DAT) showed 3+ with IgG and C3d), positive ANA with a homogenous pattern, anti-double-stranded DNA antibodies (anti-dsDNA; ELISA; titer 210 kIU/L; reference: <99 kIU/L) and complement consumption (C3 0.68 and C4 0.06 g/L, resp.; reference: 0.9–1.8 and 0.1–0.4 g/L, resp.). Antiphospholipid antibodies were absent. As such, she met the 2019 European League Against Rheumatism/American College of Rheumatology (EULAR/ACR) classification criteria, with a total score of 29 points [1]. Mediastinal lymphadenopathy was noted, and histological examination of an extirpated mediastinal lymph node demonstrated benign polyclonal lymphoproliferation. Treatment with hydroxychloroquine, glucocorticosteroids (prednisolone 60 mg once daily), and rituximab (4 × 375 mg/m^2^; indication AIHA) was initiated, resulting in remission of the mucocutaneous manifestations, serositis, and AIHA. Several years later, while being on azathioprine and low-dose glucocorticosteroid maintenance therapy, our patient developed a combined nephritic and nephrotic syndrome with preserved kidney function. Renal biopsy showed a diffuse proliferative endocapillary glomerulonephritis with subepithelial depositions and tubuloreticular inclusions and full-house immunofluorescence, consistent with classes IV + V lupus nephritis. We intensified the immunosuppression with high-dose glucocorticosteroids (prednisone 60 mg once daily) and mycophenolate mofetil (1500 mg twice daily). This led to a complete renal response, after which the patient continued maintenance treatment with reduced doses of glucocorticosteroids (prednisolone 5 mg once daily) and mycophenolate mofetil (500 mg twice daily). During an episode of lobar pneumococcal pneumonia, we noted focal bronchiectasis, which was not visible on a computed tomography (CT) scan performed 2 years earlier (Figure 1A,B). Additional immunological analysis showed hypogammaglobulinemia (IgG 4.8, IgA 0.25, and IgM 0.05 g/L; reference: 7–16, 0.7–4, and 0.4–2.3 g/L, resp.). We considered urinary protein loss from nephrotic syndrome and/or previous treatment with rituximab as possible causes; however, the hypogammaglobulinemia persisted despite reaching remission of the nephrotic syndrome and in the presence of normal B-cell counts (0.119 × 10^9^/L; reference: 0.1–0.5 × 10^9^/L). While other immunosuppressive medications, such as glucocorticosteroids and azathioprine/mycophenolate mofetil, can be associated with hypogammaglobulinemia, they were not considered a sufficient explanation for the severe hypogammaglobulinemia in this case. Other secondary causes for secondary hypogammaglobulinemia were also ruled out, such as hematologic malignancy (based on previous lymph node extirpation, positron emission tomography–computed tomography (PET-CT) and immunophenotyping of peripheral blood) and protein-losing enteropathy (as the patient did not have diarrhea or persistent hypoalbuminemia) [2]. Vaccination responses revealed inadequate IgG responses to both tetanus toxoid and pneumovax-23 (calculated post-vaccination to pre-vaccination antibody titer ratios were 1.11 and 0.97, resp.; reference: >2). 

Additional immunophenotyping showed a remarkably small population of switched memory B-cells (0.2% of CD19^+^ B-cells; reference: >10%) and a highly differentiated CD8^+^ T-cell compartment (81% of CD8^+^ T-cells comprised effector memory CD8^+^ T-cells; reference: <5% in CMV IgG-negative patients). Impaired in vitro differentiation of B-cells into plasmablasts was found using a lymphocyte proliferation assay [3]. The combination of bronchiectasis, hypogammaglobulinemia, impaired vaccination responses, and decreased switched memory B-cells with impaired differentiation into plasmablasts is consistent with a functional humoral immune defect. The patient’s family history revealed no consanguinity. Whole-exome sequencing followed by filtration for Inborn Errors of Immunity (IEI) genes showed a pathogenic heterozygous variant in the *LPS responsive beige-like anchor protein* (*LRBA*) gene. Although this finding was not diagnostic for the autosomal recessive disease LRBA deficiency, we confirmed its clinical relevance by demonstrating a strongly reduced cytotoxic T-lymphocyte-associated protein 4 (CTLA-4) expression on the T-cells (Figure 1C) [4]. Currently, we are performing additional genetic testing to demonstrate a second allelic mutation. We diagnosed the patient with LRBA deficiency, which is classified as a ‘regulatory T-cell defect’ by the International Union of Immunological Studies (IUIS) [5]. This disease causes enhanced T-cell activation and secondarily impaired B-cell activation, which manifests clinically with a humoral immunodeficiency and immune dysregulation that can result in features of autoimmune diseases such as SLE, as is apparent in this case. We started our patient on antibiotics ‘on demand’, as she refused intravenous immunoglobulin (IVIG) supplementation. In the further course of her disease, our patient developed a renal flare, AIHA recurrence, and autoimmune hepatitis, after which we started targeted therapy in the form of abatacept (CTLA-4 Ig), which can partly correct the clinical features of the genetic defect of the patient [6].

**Figure 2:**
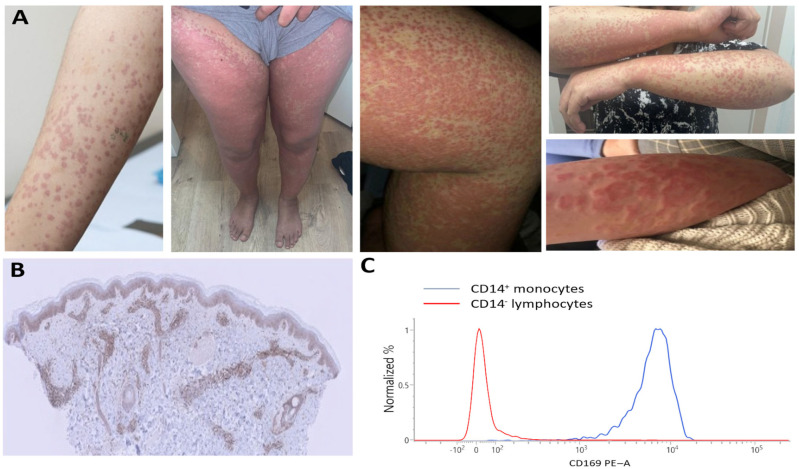
(**A**) Inflammatory skin lesions showing vasculitis in hive-like rash. (**B**) Positive myxovirus resistance protein 1 (MxA) staining in dermatocytes using MxA immunostaining, with original magnification ×10. (**C**) Increased CD169 expression on CD14^+^ monocytes demonstrated through flow cytometry.

Case 2: Our second patient is a 22-year-old Egyptian woman presenting with a constellation of symptoms, including Raynaud’s phenomenon, inflammatory arthralgia, mild sicca complaints, oral ulcers, chilblain lupus, non-scarring alopecia, and lymphadenopathy. Laboratory investigations revealed normocytic (non-hemolytic) anemia, leukopenia with lymphopenia, elevated C-reactive protein, and an elevated erythrocyte sedimentation rate, alongside polyclonal hyper-IgG (with normal IgA and IgM). Her ANA test was positive (speckled pattern; titer: 1:3200 kIU/L; reference: <99 kIU/L), anti-dsDNA and anti-Sm antibody tests were negative, and anti-SSA was positive. Complement levels (C3, C4, and C1q) were normal, and antiphospholipid antibodies were absent. Sjögren’s disease was excluded by a negative Schirmer test, leading to the diagnosis of SLE. She met the 2019 EULAR/ACR classification criteria, with a total score of 11 points [1]. She also experienced episodes of a generalized hive-like skin rash and purpura (Figure 2A) accompanied by malaise and a subfebrile temperature. 

Skin biopsies showed leukocytoclastic vasculitis with isolated C3 deposition. Given her normal complement levels, absence of anti-C1q antibodies, and lack of a monoclonal protein, we concluded the hives were unlikely due to the SLE-associated urticarial vasculitis syndrome or Schnitzler syndrome. The patient showed no clinical response to treatment with hydroxychloroquine (200 mg once daily), prednisone (20 mg once daily), azathioprine (150 mg once daily), and cyclosporine (150 mg twice daily). Further investigation revealed positive myxovirus resistance protein 1 (MxA) staining on skin biopsies, a positive interferon signature, and increased CD169 expression on monocytes (Figure 2B,C), all suggesting that type I interferons played a significant role in her disease. In SLE pathogenesis, interferon overproduction has been recognized to be a key feature in hyperactivating the innate and adaptive immune system. However, interferon overproduction is also a central feature in monogenic interferon-mediated diseases. This, together with the refractoriness of her symptoms to treatment, led us to consider monogenic interferon-mediated diseases. Whole-exome sequencing followed by filtration for IEI genes demonstrated a bi-allelic pathogenic variant in *RNASEH2C*, consistent with Aicardi–Goutières syndrome (AGS). Despite two epileptic episodes, our patient exhibited no neurological abnormalities on examination or cerebral MRI. Furthermore, there was no evidence of glaucoma or cardiomegaly/cardiomyopathy, which are findings associated with *RNASEH2C*-AGS. Together with the absence of young-onset disease, this underscores the disease’s variable penetrance. We initiated family screening and started treatment with JAK inhibition, given its suppressive effect on interferon-dependent genes through blockading the JAK-STAT pathway, followed by interferon-receptor blockade (anifrolumab). However, neither treatment showed a clinical effect over a six-month period. The systemic inflammation and cutaneous vasculitis are currently being managed with anti-interleukin-1 blockade.

**Figure 3:**
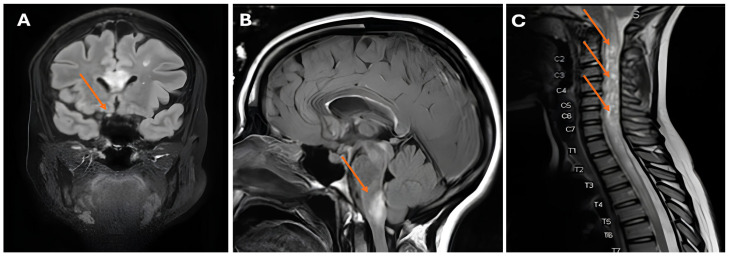
(**A**) Abnormal appearance and pathological enhancement of the right optic nerve (arrow); (**B**) white matter lesions in the brachium pontis (arrow); (**C**) longitudinally extensive spinal cord lesion with swelling and the appearance of bright spotty lesions (arrows).

Case 3: Our third case is a woman diagnosed with SLE at 28 years old based on polyarthritis, a malar rash, a positive ANA test (homogenous pattern), and elevated anti-dsDNA antibodies (ELISA; titer 2290 kIU/L; reference: <99 kIU/L), as well as positive anti-Sm antibody tests (anti-SSA, anti-SSB, and anti-Ro52). Her complement C3 levels were lowered (0.65 g/L; reference: 0.9–1.8 g/L), with normal C4 levels. She experienced several thrombotic events, including a pulmonary embolism, cerebral venous sinus thrombosis, and Libman–Sacks endocarditis. These occurred in the setting of secondary antiphospholipid syndrome, confirmed by the consistent presence of lupus anticoagulant (LAC), anti-β2-glycoprotein I (aβ2GP; titer IgG 23 U/mL; reference: 0–7 U/mL), and anti-cardiolipin antibodies (aCL; IgG 65 U/mL; reference: 0–10 U/mL), for which anticoagulative therapy with a vitamin K antagonist was started. She met the 2019 EULAR/ACR classification criteria, with a total score of 21 points [1]. At age 38, she suffered from acute paresis of her right arm and leg, along with nausea and headache, which then progressed to aphasia. Neurological examination identified right-sided hemiparesis and a positive Babinsky sign. A cerebral MRI showed acute disseminated encephalomyelitis (ADEM spectrum). We considered the neurologic manifestations to be compatible with a major flare of her SLE, and we initiated treatment with MPNS pulses (1 g for 3 consecutive days) and cyclophosphamide (once monthly according to the BSA schedule [7]), and our patient recovered with mild cognitive impairment. Her maintenance treatment consisted of low-dose glucocorticosteroids (prednisolone 5 mg once daily) and mycophenolate mofetil (500 mg twice daily). She experienced no flares in subsequent years. However, at age 43, she presented with partial paraplegia and multiple white matter lesions in her spinal cord. Diagnostic evaluation of liquor demonstrated pleocytosis with negative serology and molecular tests for infectious pathogens. We initiated induction treatment with MPNS pulses (1 g for 3 consecutive days) and cyclophosphamide (500 mg every 2 weeks for 6 consecutive cycles according to the Euro-Lupus schedule [8]) under clinical suspicion of a major SLE flare. Following the second dose of cyclophosphamide, the patient developed right-sided optic neuritis with a severe decline in visual acuity (Figure 3). Given that the patient’s neurologic symptoms were both refractory to initial SLE induction treatment and more extensive than is typical for SLE, serological testing for anti-aquaporin-4 (AQP4-IgG) and anti-myelin oligodendrocyte glycoprotein (MOG-IgG) antibodies was performed. The anti-AQ4 antibodies were positive, consistent with the concurrent diagnosis of clinical isolated subtype of neuromyelitis optica spectrum disorder (NOMSD). We switched her treatment from cyclophosphamide to rituximab (2 × 1000 mg), which resulted in clinical improvement. Although NOMSD can be a feature of SLE, in this case, NOMSD presented as a concurrent disease, which rendered a different treatment approach.

## 3. Discussion

We present three cases—two initially diagnosed as SLE and one as an SLE flare—where the diagnosis was revised at different points in the disease course. Although the clinical presentations of these cases were highly diverse, several overarching lessons can be learned. We discuss these lessons and review the literature for SLE-mimicking diseases.

In our initial case of IEI, it is remarkable that the patient presented with typical clinical, biochemical, serological, and histological features consistent with SLE. However, diagnostic clues suggesting a differential diagnosis were present from the moment of presentation. First, analysis of AIHA revealed a DAT with a predominance of cold (C3d) over warm (IgG) antibodies, which is atypical for SLE-associated AIHA [9]. This finding prompted a PET-CT scan, which revealed mediastinal lymphadenopathy with histological evidence of polyclonal hyperplasia. Initially, after excluding lymphoproliferation and infections, this was attributed to SLE lymphadenopathy [10]. However, the presence of cold agglutinins in association with polyclonal hyperplasia can also suggest an underlying IEI. It was not until a hospital admission for lobar pneumococcal pneumonia that we noted new-onset bronchiectasis and hypogammaglobulinemia. The hypogammaglobulinemia was not attributed to renal protein loss, as the nephrotic syndrome was in sustained remission, nor was it explained by rituximab treatment, as post-treatment B-cell reconstitution had occurred. Nonetheless, we cannot entirely exclude the potential impact of immunosuppression use on the immunological work-up. Crucially, the detection of a heterozygous pathogenic variant in *LRBA*, with functional confirmation of loss of CTLA-4 expression on regulatory T-cells, provided strong evidence of an underlying IEI [11]. The genetic diagnosis for this autosomal recessive disease requires confirmation by finding a second mutant allele for which a fibroblast cell line is developed to perform cDNA analysis. While monoallelic expression is a growing area of study in IEI and other monogenic diseases [12], it has not been described in LRBA deficiency. It is well-established that IEIs can manifest with autoimmunity and autoinflammation, sometimes even preceding the onset of infections [13,14]. There may also be lymphoproliferation and allergy [15,16]. This can make the timely recognition of an underlying IEI in a patient with immune dysregulation difficult, where studies show a diagnostic delay of many years [17]. *LRBA* regulates the intracellular recycling of the CTLA-4 receptor on T-cells; its loss of expression leads to T-cell activation and secondary impaired B-cell activation, proliferation, and subsequent differentiation into antibody-producing plasma cells [4]. This results in vulnerability to upper airway infections by encapsulated pathogens, as well as autoimmunity. LRBA deficiency can present with a phenotype resembling SLE, making it a crucial differential diagnosis [18]. 

In our second case of monogenic interferonopathy, the patient presented with several features that overlapped significantly with SLE, including Raynaud’s phenomenon, mucocutaneous involvement, anemia, leukopenia, lymphopenia, and a positive ANA test. However, key distinctions were also present. Atypical findings for SLE included markedly elevated inflammatory markers and a non-homologous ANA pattern, though ANA patterns can be variable across different populations [19,20]. The absence of anti-dsDNA and anti-Sm antibodies and a lack of complement consumption, while unusual for SLE, do not exclude the diagnosis [1]. It is noteworthy that an urticarial rash, as seen in this patient, has been described in SLE patients with urticarial vasculitis and anti-C1q antibodies [21]. The substantial clinical overlap between hypocomplementemic urticarial vasculitis and SLE is well documented, with approximately 50% of patients with the former eventually receiving an SLE diagnosis [21]. The combination of treatment refractoriness and consanguinity prompted us to consider a genetic cause for the observed type I interferon pathway activation, even though this activation is also a known hallmark of SLE and other autoimmune diseases [22]. This led to genetic testing and a subsequent AGS diagnosis, which is a prototypical monogenic type I interferonopathy. Defined by nine genotypes (AGS 1-9), AGS is caused by pathogenic variants in genes essential for nucleic acid processing and signaling [23]. The pathogenesis involves dysregulated DNA- or RNA-mediated signaling, causing a breakdown in the discrimination between ‘self’ and ‘non-self’ nucleic acids, resulting in a persistent, elevated production of type I interferons [24]. Interestingly, our patient did not exhibit classic AGS features, such as young-onset neurodevelopmental delay, spasticity, intracranial calcification, or cerebral atrophy [25]. Furthermore, other commonly associated features, such as cardiomyopathy and glaucoma, were absent. This finding highlights the significant clinical variability and incomplete penetrance of monogenic diseases, suggesting that polygenic and epigenetic factors may modulate the genotype–phenotype relationship [26]. The growing accessibility of genetic testing continues to reveal these diverse and often uncharacteristic disease presentations. Ultimately, this case underscores a crucial diagnostic approach: in patients with both autoimmune features and evidence of type I interferon activation, physicians must consider not only common systemic autoimmune diseases but also rare monogenic interferonopathies, particularly in the context of consanguinity. While markers of type I interferon activation, such as ISG, MxA, and CD169, are increasingly being explored for diagnostic and therapeutic guidance in unclassified systemic diseases [27,28,29,30], their routine clinical use requires further validation.

The third patient in our series was diagnosed with SLE and secondary antiphospholipid syndrome, a conclusion substantiated by her compatible clinical phenotype and serologic findings. Her initial neurological manifestation of ADEM, although rare, is compatible with SLE [31] and responded to conventional treatment. Five years later, the patient experienced clinical deterioration that was refractory to induction treatment. Furthermore, the development of a pattern characteristic of a distinct disease entity—specifically, clinically isolated NMO, as evidenced by bilateral optic neuritis (with a severe decline in visual acuity) and multifocal cerebral lesions—compelled us to reconsider an SLE flare diagnosis. Upon positive testing for serum AQP4-IgG, the diagnosis NMO was added to the existing SLE diagnosis. Demyelinating syndromes in SLE patients present with varied phenotypes. It is essential to distinguish between those that represent an SLE flare and those that constitute a concomitant, isolated disease (e.g., clinically isolated NMO [32,33]), as observed in our patient. This differentiation has significant clinical relevance, directly impacting treatment decisions. Our case specifically highlights the importance of not attributing all new manifestations to SLE, demonstrating that they may originate from a concurrent disease, such as isolated NMO.

SLE is known for its wide range of manifestations, potentially affecting any organ system. When assessing a patient for the diagnosis of SLE, clinicians should consider other conditions that share similar characteristics. It is crucial to remember that SLE classification criteria are designed for research purposes, aiming for homogeneous patient groups [1]. While these criteria have a relatively high specificity, they are not designed to serve as a diagnostic tool in individual clinical cases [1]. Several factors should prompt a clinician to re-evaluate an SLE diagnosis. These include atypical disease presentation, inconsistent serology, and lack of treatment response. Further complicating the diagnostic challenge, a positive ANA, though a hallmark of SLE, can be clinically insignificant in 5–10% of the general population aged 60 and older [34]. In considering the relevance of a positive ANA, a diligent diagnostic workup must exclude other potential causes, especially in cases with atypical features. Infections, such as Parvovirus B19, can mimic SLE by inducing a positive ANA and immunofluorescence-positive immune deposits [35]; therefore, excluding them is essential before initiating immunosuppressive therapy. Furthermore, drug-induced lupus should be considered in patients on medication; discontinuing the suspected drug and retesting for the presence of ANA can help confirm or rule out this possibility [36]. The diagnostic work-up must also account for potential underlying malignancies, including lymphomas [37]. Lastly, monogenic causes presenting with SLE-like features, as illustrated by cases 1 and 2, warrant special attention [5]. A positive family history, particularly with consanguinity, strongly suggest a genetic etiology. While not exhaustive, Table 1 provides an overview of various conditions that can mimic SLE, underscoring the broad spectrum of SLE symptoms and its diverse presentations.

This case series underscores the need for diagnostic vigilance when evaluating complex autoimmune presentations, even those initially compatible with SLE. We highlight two crucial lessons: first, the necessity of considering monogenic causes, such as primary immunodeficiencies and monogenic interferonopathies, which mimic SLE, particularly in cases with atypical features or refractory disease; second, the importance of distinguishing between an SLE flare and a coexisting, distinct pathology, as illustrated by the diagnosis of clinical isolated NMO. Clinicians must recognize the inherent limitations of SLE classification criteria as a sole diagnostic tool and maintain a broad differential diagnosis. Ultimately, the presence of unusual clinical, serologic, or treatment response patterns should prompt a dynamic, multi-faceted diagnostic reassessment, ensuring that patients receive timely, mechanism-specific therapy rather than being confined to a single, initial diagnostic label.

## Figures and Tables

**Table 1:** Overview of SLE-mimicking diseases.

**Non-SLE systemic connective tissue and autoimmune disease**
Dermatomyositis, Sjögren’s disease, mixed connective tissue disease, Still’s disease, neuromyelitis optica spectrum disease, multiple sclerosis, and Castleman disease.
**Monogenic diseases**
Interferonopathies (i.e., Aicardi–Goutières, SAVI, PRAAS, and Singleton–Merten syndrome).
Complement deficiencies (i.e., c1q/c1s/c1r/c2/complete C4 deficiency).
Other Inborn Errors of Immunity (i.e., HELIOS deficiency, SOCS1 haploinsufficiency, TLR7 deficiency, UNC93B1 deficiency, IRE1α deficiency, DOCK11 deficiency, autoimmune lymphoproliferative disorder, DNASE1L3 deficiency, and SPENCD).
**Infections**
Parvovirus B19, endocarditis, hepatitis, HIV, EBV, CMV, Borrelia, toxoplasmosis, histoplasmosis, mycobacterial diseases, leishmaniasis, and Whipple’s disease.
**Malignancies**
Angioimmunoblastic T-cell lymphoma, other lymphomas, and myelodysplastic syndrome.
Solid malignancies w/wo paraneoplastic features.
Atrial myxoma.
**Miscellaneous**
Drug-induced lupus.
Graft-versus-host disease.

## Data Availability

The data is available upon request from the authors.
